# Angiosarcoma of the scalp

**DOI:** 10.4103/0970-0358.53023

**Published:** 2009

**Authors:** Manjiri DasGupta, Nilay Chakrabarti, Pravin Agrawal, Swati Narurkar

**Affiliations:** Department of Surgery, K.J. Somaiya Medical College and Hospital, Sion, Mumbai - 400 022, India; 1 Department of Pathology, K.J. Somaiya Medical College and Hospital, Sion, Mumbai - 400 022, India

**Keywords:** Angiosarcoma, Malignant angioendothelioma, Malignant hemangioendothelioma of scalp

## Abstract

Angiosarcoma is a relatively rare soft tissue tumour. It usually occurs in the head and neck, and especially in the scalp, in elderly people. Its presentation varies from a small plaque to multifocal nodules. The treatment depends on the extent of the disease. Most cases are treated with wide excision with reconstruction. Radiotherapy and chemotherapy are advocated in the recurrent or extensive lesions with regional or distant metastasis. Other modalities such as immunomodulation have been tried. A case of a 55-year-old female patient with a bleeding scalp lesion is presented. Initially thought to be a pyogenic granuloma, on excisional biopsy it was diagnosed as angiosarcoma with microscopic involvement of the margins. Wide excision with reconstruction using a local rotation flap was done at a second stage. The patient was not given postoperative radiotherapy or chemotherapy. There has been no recurrence for two years.

## INTRODUCTION

Angiosarcoma is a rare soft tissue sarcoma usually seen in the head, face and neck. Five per cent of the soft tissue sarcomas occur in the head, face and neck of which 10% comprise angiosarcomas.[1,6,7,11] Angiosarcoma involving the scalp of old patients was first described as a distinct subgroup by Wilson-Jones and is usually limited to the skin and soft tissues. While 10% of these lesions develop in patients with chronic lymphoedema, another important predisposing factor is exposure to radiation. Most lesions are not associated with any pre-existing condition.[15] The disease occurs in the dermis and presents as single or multiple bluish or red nodules or plaques which ulcerate or bleed. Metastasis to regional lymph nodes or lungs can occur.[11,15] Microscopically, the tumour can vary from differentiated to poorly differentiated types and may occasionally involve deeper structures. Treatment of these lesions is usually by radical excision and subsequent reconstruction.[5,11,14] Radiotherapy and chemotherapy have also been used in unresectable tumours, or those with distant metastasis. The prognosis of such patients is very poor.

## CASE REPORT

A 55–year-old female patient presented to the outpatient department with profuse bleeding from a scalp swelling discovered during combing of her hair. The swelling had bled similarly about four to five weeks ago. However, it had now rapidly increased in size over the last week and was bleeding more frequently, even with minor friction.

**Figure 1:**
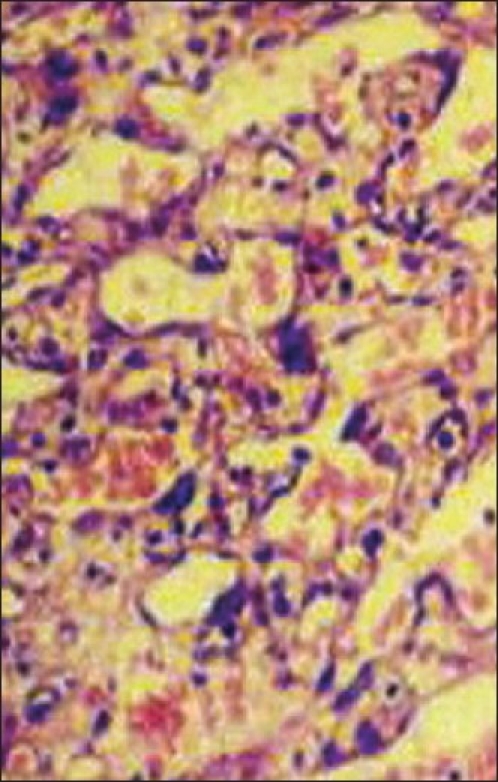
Photomicrograph of the excised lesion (H&E, ×400)

On examination, a 2 cm × 1.5 cm × 1 cm lesion was seen over the right parietal region of the scalp. It was brownish black in colour, firm in feel with irregular margins and started bleeding even as it was being palpated. The rest of the scalp was normal. There were no palpable lymph nodes in the neck. Excision of the lesion with primary closure was performed under local anaesthesia on the same day with a provisional diagnosis of infected scalp granuloma, and the specimen sent for biopsy. Histology, however, revealed dissecting and anastomosing vascular channels with focal mild cytological atypia in the dermis present beneath ulcerated and inflamed squamous epithelium. Secondary haemorrhagic infarction and necrosis were also noted. Histologically, features were consistent with a vasoformative neoplasm involving the dermis, a cutaneous angiosarcoma of the scalp, with involvement of the excised margin on one side [Figure 1].

**Figure 2:**
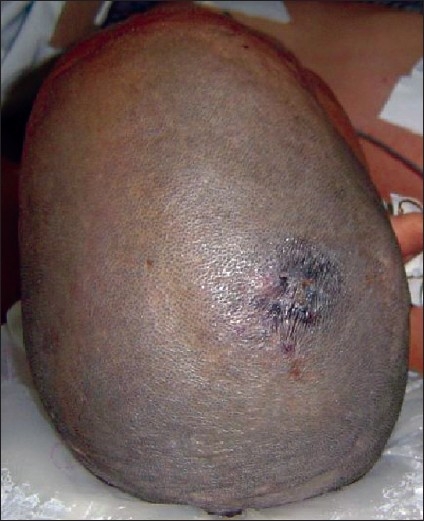
Pre-revision surgery

Since histopathological examination of the excised margin on one side showed involvement, revisional surgery in the form of a wide excision of the scar [Figure 2] was then planned after presence of distal metastasis was ruled out by an X-ray chest and abdominal ultrasonography. Wide excision of the scar was performed with a margin of three centimetres from the visual border of the scar. The pericranium was included in the base of the resected specimen and the resultant defect measured 7 cm × 6 cm. As the bone was exposed, reconstruction was performed with a local rotation flap. A negative suction drain was inserted below the flap prior to closure.

**Figure 3:**
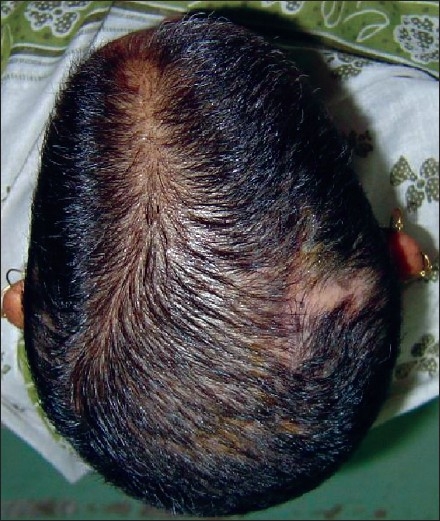
Post-revision view

Sutures were removed on the ninth postoperative day and the wound healed well [Figure 3]. Histopathology of the excised specimen showed the margins to be well clear of the lesion on this occasion. The patient was not administered any adjuvant therapy. She was followed up every six months and has had no recurrence after two years.

## DISCUSSION

Angiosarcoma is a rare soft tissue sarcoma arising from the endothelial cells varying from well-differentiated tumours to poorly differentiated ones. Microscopically, they involve the dermis while the poorly differentiated ones may invade into deeper structures. Low-grade angiosarcomas are well differentiated while the high-grade lesions are poorly differentiated and consist of sheets of pleomorphic cells with areas of haemorrhage, disordered architecture, cells with hyperchromatic and pleomorphic nuclei with prominent mitotic activity. Both types are associated with extensive local growth. The grade of the tumour, however, does not have any correlation with survival.[5,11] Cutaneous angiosarcomas may be associated with chronic lymphoedema, previous radiation therapy, treatment of breast carcinomas and immunosupression in renal transplant patients.[1–3,5,11,14] The angiosarcoma following treatment of breast carcinoma was attributed to chronic lymphoedema but recently with the advent of breast-conserving surgery, the incidence of lymphoedema has reduced. However, a form of cutaneous, post-radiation angiosarcoma of the breast (CPRSAB) has still been described.[2] There have been reports of a series of radiation-induced angiosarcomas in which the time interval between the exposure and development of angiosarcoma was six years.[3] The basis of development of angiosarcoma in these patients has been postulated to be radiation-induced connective tissue damage.[3] Exposure to the sun and the resultant actinic skin damage was proposed as a cause of angiosarcoma due to its predominance in Caucasians and rarity in the coloured races. This theory however was refuted by the fact that most patients with angiosarcoma of the scalp had hair which gives protection from exposure to the sun.[8] Other predisposing factors that are reported include occurrence of tumour in previous herpes zoster sites, telangiectating nevus, other vascular and lymphatic abnormalities, arterio-venous fistula, chronic osteomyelitis, and exposure to arsenic, thorotrast and polyvinyl chloride.[5,7,11,14] Trauma usually alerts the patient to the presence of the lesion and is not responsible for the development of angiosarcoma. However, it cannot be denied that in most patients no underlying predisposing factor is found. In our patient there was no identifiable predisposing factor. Angiosarcomas occur commonly in Caucasians, with very few cases reported in other races. Due to their rare occurrence and common resemblance to benign conditions, these lesions are often diagnosed late. Angiosarcoma of the scalp occurs in elderly male patients 68 to 76 years old with an overall male-to-female ratio of 2:1.[1,7,11,14] Our patient however was a female in the mid- fifties. Most patients present with a bruise-like macule or a non bruise-like nodule.[11] Other common presentations include indurated, erythematic nodules, fungating masses, ulcerations or sometimes as bleeding lesions, as in our patient. Ulcerated, fungating and haemorrhagic lesions indicate advanced disease while unusual presentations too have been reported. Nkamura *et al.*, have reported a diffuse widespread angiosarcoma of the scalp presenting with Kasabach-Merritt phenomenon with consumption coagulopathy and thrombocytopenia which resolved only with the regression of the tumour[9] while Knight *et al.*, have reported a rare case where the angiosarcoma of the scalp presented as extensive scarring alopecia.[12] It has the highest rate of lymph node metastases among all soft tissue sarcomas of the head and neck and distant metastasis may occur in up to 50% with the lung being the most common site followed by liver.[8] Delayed recurrence of angiosarcoma at the distant sites has been reported, which makes regular, lifelong surveillance a must. The highly metastatic nature of these tumours has been attributed to the absence of vascular endothelial cadherin (VE-cadherin), which is present in the normal endothelium. In soft tissue sarcomas, the statistically significant predictors of subsequent metastasis include size of the tumour, its grade and depth including neurovascular or bone involvement. Except for the tumour grade all the other factors depend upon the tumour size. Therefore Obeng *et al.*, have emphasized on the need for early diagnosis and aggressive management which includes wide tumour excision.[8] Multifocal disease is associated with a shorter interval between initial presentation and recurrence.[11] Younger patients have better prognosis[11,14] while presence of metastasis at the initial presentation is associated with poor prognosis. Overall prognosis is reported to be very poor, the five-year survival being less than 10-30%.

Cutaneous angiosarcoma is difficult to treat due to its multicentric occurrence and the presence of extensive microscopic spread that is very common in these tumours. The treatment described is wide excision of the lesion to achieve histologically tumour-free margin as this has a direct impact on the prognosis.[5,8,11,14] As the microscopic spread of the tumour is extensive, primary closure of the wound is often not possible after wide excision. A staged reconstruction is performed only after confirming tumour-free margins on histopathological examination. Various reconstructive options are available such as split-thickness skin grafts, local flaps and free flaps. As these patients are elderly, the preferred reconstructive algorithm used is skin grafts, local flaps and free flap.[14] The skin grafts are the commonest except when the excision includes the pericranium or when there is history of previous radiotherapy.[14] Local rotation flap is indicated when the pericranium is also excised and the defect is not extensive. When the angiosarcoma is multicentric and extensive, it may require excision of the whole scalp. Such an extensive defect is reconstructed with free flaps. Postoperative low-dose, very wide-field radiation is effective in treating local disease following resection of clinically evident tumour.[9] Radiation is also indicated in patients with diffuse multifocal lesions. Improved survival rate with routine use of postoperative radiotherapy is seen in only 21% patients who had tumour-free margins.[11] Use of high-dose brachytherapy with a surface mould technique to avoid marginal recurrence common with conventional technique, in extensive angiosarcoma of the scalp, has also been reported.[10] Other modalities such as cytokine therapy have been reported in the form of intralesional interferon alpha-2b and interleukin-2 combined with surface radiotherapy, as an alternative to surgery.[13] The role of conventional chemotherapy is debatable since angiosarcoma is a rare disease for which there is no established chemotherapy. However, use of liposomal doxorubicin with radiotherapy, in a wide lesion, has been reported.[15] Recent advances in the treatment of angiosarcomas include the use of Placlitaxel. Placlitaxel has been reported to be an active agent against angiosarcoma of the scalp due to its antiangiogenic properties by researchers at the Memorial Sloan-Kettering Cancer Center, New York.[4] In our case we have treated the patient with wide excision only. As the surgical margins were free of tumour on histopathology after wide excision, we did not use postoperative radiotherapy or chemotherapy.

## CONCLUSION

Angiosarcoma of the scalp is a very aggressive tumour with poor prognosis. Outcome can be improved with early diagnosis and aggressive early treatment. In all elderly patients with lesions on the scalp and head and neck region, the possibility of angiosarcoma should be kept in mind to avoid unnecessary delay in treatment. Wide surgical excision to achieve tumour-free margin is associated with improved survival. Widespread lesions where tumour-free margins cannot be achieved, or patients with local recurrences and metastasis, need other modalities. Lifelong surveillance is advised to detect any delayed distant metastasis.
